# A decrease in integrin α5β1/FAK is associated with increased apoptosis of aortic smooth muscle cells in acute type a aortic dissection

**DOI:** 10.1186/s12872-024-03778-2

**Published:** 2024-03-26

**Authors:** Mingming Xue, Lingyu Xing, Yilin Yang, Mian Shao, Fengqing Liao, Feixiang Xu, Yumei Chen, Sheng Wang, Bin Chen, Chenling Yao, Guorong Gu, Chaoyang Tong

**Affiliations:** Department of Emergency Medicine, Zhongshan Hospital, Fudan University, Shanghai, 200032 China

**Keywords:** Acute type a aortic dissection, Integrin α5β, Human aortic smooth cell, Apoptosis

## Abstract

**Background:**

Acute type A aortic dissection (AAAD) is a devastating disease. Human aortic smooth muscle cells (HASMCs) exhibit decreased proliferation and increased apoptosis, and integrin α5β1 and FAK are important proangiogenic factors involved in regulating angiogenesis. The aim of this study was to investigate the role of integrin α5β1 and FAK in patients with AAAD and the potential underlying mechanisms.

**Methods:**

Aortic tissue samples were obtained from 8 patients with AAAD and 4 organ donors at Zhongshan Hospital of Fudan University. The level of apoptosis in the aortic tissues was assessed by immunohistochemical (IHC) staining and terminal-deoxynucleotidyl transferase-mediated nick end labeling (TUNEL) assays. The expression of integrin α5β1 and FAK was determined. Integrin α5β1 was found to be significantly expressed in HASMCs, and its interaction with FAK was assessed via coimmunoprecipitation (Co-IP) analysis. Proliferation and apoptosis were assessed by Cell Counting Kit-8 (CCK-8) assays and flow cytometry after integrin α5β1 deficiency.

**Results:**

The levels of integrin α5β1 and FAK were both significantly decreased in patients with AAAD. Downregulating the expression of integrin α5β1-FAK strongly increased apoptosis and decreased proliferation in HASMCs, indicating that integrin α5β1-FAK might play an important role in the development of AAAD.

**Conclusions:**

Downregulation of integrin α5β1-FAK is associated with increased apoptosis and decreased proliferation in aortic smooth muscle cells and may be a potential therapeutic strategy for AAAD.

**Supplementary Information:**

The online version contains supplementary material available at 10.1186/s12872-024-03778-2.

## Introduction

Acute type A aortic dissection (AAAD) is a life-threatening condition with a mortality rate of 1 to 2% per hour after symptom onset in untreated patients [1, 2]. Surgical repair is the main treatment for patients who present with AAAD [3, 4]. Integrins are heterodimeric cell surface receptors. These molecules are important regulators of the mechanical engagement of the cell with the extracellular matrix [5]. Integrins can transmit intracellular and extracellular signals in various diseases, including cardiovascular diseases, malignant tumors and autoimmune diseases. Integrin α5β1 is a heterodimer composed of the α5 and β1 subunits and is a proangiogenic factor involved in regulating angiogenesis [6]. Cells of the aortic wall, such as endothelial cells (ECs), vascular smooth muscle cells (VSMCs) and platelets, express cell type-specific integrins under healthy and diseased conditions. The hyperexpression of α5β1 integrin in VSMCs has been shown to promote the metastasis of several types of cancer, such as lung cancer [7, 8] and melanoma [9, 10]; however, α5β1 integrin also has a tumor-suppressive effect in breast cancer [11–13] and colon cancer cell lines [14, 15]. The alterations in integrin α5β1 in patients with AAAD are unknown.

In this study, we found that the expression of integrin α5β1 was decreased in human aortic smooth muscle cells (HASMCs) from surgical specimens of AAAD. To determine whether the integrin α5β1 pathway is involved in the pathogenesis of aortic dissection, we used siRNAs to target integrin α5 and integrin β1 in HASMCs. We found that HASMC apoptosis was significantly increased, and integrin α5β1 directly bound to and reduced the phosphorylation level of FAK, resulting in aortic SMC apoptosis.

## Materials and methods

### Human aortic tissue specimens

Of the 12 aortic samples, 4 were from heart transplant donors and 8 were obtained in the operating room from patients with acute aortic dissection who underwent an aortic replacement operation. The clinical and genetic characteristics of the enrolled patients are shown in Supplementary Table 1 (a, b). Aortic specimens were taken from the hematoma. Aortic fragments from the same region taken from healthy donors during the organ transplantation procedure were used as the negative controls. The specimens were immediately placed in PBS on ice and transported to the laboratory for further processing. Each specimen was divided into two parts. One half was snap-frozen in liquid nitrogen and stored at -80 °C until subsequent use. The tissue was fixed in a 4% paraformaldehyde solution and embedded in paraffin for 12 h. The other half of the specimen was subjected to immunohistochemical (IHC) or immunofluorescence (IF) staining. The study was approved by the Ethics Committee of Zhongshan Hospital, and informed consent was obtained from the close relatives of the participants. The study was conducted following the principles of the Declaration of Helsinki.

### Cells

Cultured 6110 HASMCs and smooth muscle cell medium (SMCM) 1101 were purchased from ScienCell™ Research Laboratory (USA). The cells were cultured according to the manufacturer’s instructions.

### Materials

Ab150361 (human anti-integrin α5 antibody), Ab179471 (human anti-integrin β1 antibody), and anti-VEGF receptor 2 were purchased from Abcam (USA). Ab3281 [phospho-FAK (Tyr576/577) antibody] was purchased from Cell Signaling (USA). 66258-1-Ig (FAK monoclonal antibody), 19677-1-AP (caspase 3 antibody), 60267-1-Ig (Bax monoclonal antibody), 60178-1-Ig (Bcl2 monoclonal antibody), and 60004-1-1 (GAPDH antibody) were purchased from Proteintech (USA). Alexa Fluor 488 AffiniPure goat anti-mouse IgG (33206ES60) and Alexa Fluor 594 AffiniPure goat anti-rabbit IgG (33112ES60) were purchased from Yeasen (China).

All the other chemicals and reagents used were purchased from Sigma (USA). All reagents were used according to the manufacturers’ instructions.

### Immunohistochemistry

IHC staining for integrin α5, integrin β1, p-FAK, and VEGFR2 was conducted according to the following procedures. The paraffin-embedded tissue samples of 8 patients with AAAD from Zhongshan Hospital were heated at 60 °C for 4 h and deparaffinized. The slides were then immersed in 3% H_2_O_2_ to inactivate endogenous peroxidase. The tissues were incubated with 1% BSA to block nonspecific binding and incubated at 4 °C overnight. The next day, the slides were exposed to horseradish peroxidase-linked secondary antibody for 1 h. Finally, the slides were developed for signal detection using 0.05% 3,3’diaminobenzidine (DAB) and counterstained with hematoxylin to achieve optimal staining intensity.

### Terminal-deoxynucleotidyl transferase-mediated nick end labeling (TUNEL) analysis

The paraffin-embedded tissue samples from 8 patients with AAAD were deparaffinized and incubated in distilled water and PBS for 5 min. Antigen repair was performed with protease K working solution. The tissues were incubated with 1% BSA for 30 min, after which TDT enzyme reaction solution was added. The slides were covered and incubated with antibodies at 4 °C overnight. The next day, the slides were exposed to secondary antibody and TUNEL test solution, incubated at 37 °C in the dark for 60 min, and then washed in PBS 3 times. After the membrane was sealed with antifluorescence quenching sealing solution, it was observed by fluorescence microscopy.

### Western blot

Total protein was extracted from aortic tissue and human aortic smooth cells using RIPA lysis buffer (Sangon Biotech, China) according to the manufacturer’s instructions. A BCA protein assay kit (Sangon Biotech, China) was used to determine the protein concentration in the lysates. After electrophoresis, the membranes were treated with specific antibodies overnight at 4 °C and then incubated with the following secondary antibodies: anti-integrin α5 antibody (ab150361, Abcam), anti-integrin β1 antibody (ab17947, Abcam), FAK monoclonal antibody (66258-1-Ig, Proteintech), anti-VEGF receptor 2 antibody (ab2349, Abcam), phospho-FAK (Tyr576/577) antibody (3281, Cell Signaling), and GAPDH antibody (60004-1-1, Proteintech). An enhanced chemiluminescence (ECL) assay kit (36208ES76, Yeasen, China) was used to assess the proteins.

## IF

Small discs were placed in a medium dish and soaked in 100% ethanol for disinfection. The small disc was then placed on a 24-well plate, washed with PBS 3 times, and then washed with culture solution 3 times. The cell volume was 50–60% 24 h after plating. After 24 h of cell growth, the plate was removed, the culture medium was aspirated, and the cells were washed with PBS 3 times. The cells were then fixed at room temperature for 15 min, washed with PBS 3 times, and then washed with 4% paraformaldehyde. Next, 1% BSA was added, and the cells were blocked at room temperature for 30 min. The primary antibody diluted in 1% BSA was then added, and the mixture was incubated overnight at 4 °C. The next day, the sample was washed with PBS 3 times, diluted fluorescent secondary antibody was added, and the sample was incubated at room temperature in the dark for 60 min, followed by another 3 washes with PBS. For DAPI staining, DAPI solution was added, and the entire sample was incubated at room temperature in the dark for 10 min and then washed with PBS. After the addition of an antifluorescence quenching agent, the membrane was sealed, and the slides were observed and photographed under a microscope.

### qRT–PCR

Total RNA was extracted from cultured aortic SMCs using TRIzol reagent (15,596,026, Invitrogen, Carlsbad, CA, USA). The specific sense and antisense primers for integrin α5 were 5′-CAGCCTTGCCAGAGATCCAA-3′ and 5′-TCCTTGTGTGGCATCTGTCC-3′, respectively; those for integrin β1 were 5′-TGCAACAGCTCTCACCTACG-3′ and 5′-ACAGTGGTCTGTTATGGCACT-3′, respectively. Briefly, 10 µL of solution containing 1 µg of total RNA, 10 µL of 2x SYBR Green I reagent, 4 U of Multi-Scribe reverse transcriptase, 5 U of RNase inhibitor and 0.5 mM primers was subjected to one cycle of 50 °C for 2 min and 95 °C for 10 min and then 40 cycles of 95 °C for 15 s and 60 °C for 1 min. mRNA transcripts were first normalized to those of the control integrin α5 or integrin β1. The differential expression of these genes was subsequently analyzed by the ΔΔCt method, and the results are shown as fold changes.

### Cell counting Kit-8 (CCK-8) assay

CCK-8 kits (C0037) were purchased from Beyotime (China). The digested cells were inoculated with 100 µL of cells at a density of 5 × 10^3^ in a 96-well culture plate and cultured for 24 h. The next day, the cells had adhered to the wall and were transfected with integrin α5 siRNA and integrin β1 siRNA. Then, the cells were cultured in a 37 °C incubator for 48 h. Ten microliters of CCK-8 solution was added to each well. Then, the cells were cultured for 4 h. The optical density (OD) values of the cells in each well were determined at 450 nm and used to calculate the cell survival rate.

### Flow cytometry

An Annexin V-FITC cell apoptosis detection kit was purchased from Beyotime (China). Briefly, HASMCs were collected, washed twice with PBS, and resuspended in FACS buffer (PBS with 10% serum and 1% BSA). The cells (5–10 × 10^5^) were then incubated with an Annexin V monoclonal antibody at 4 °C for 30 min in the dark. Then, 10 µL of propidium iodide (PI) solution was added to stain the cells. The cells were incubated at room temperature in the dark for 10–20 min and analyzed on a BD FACSCanto II flow cytometer. Then, the cells were observed under a fluorescence microscope. Annexin V-FITC showed green fluorescence, and PI showed red fluorescence.

### Coimmunoprecipitation analysis

The HASMCs were suspended in ice-cold PBS containing protease inhibitors (Thermo Fisher Scientific). The cell lysates were centrifuged (10,000×g for 15 min at 4 °C) and collected. The protein in the cell lysate was analyzed by a BCA protein quantitative kit (Sangon Biotech, China). The cell lysate was added to the primary antibody (1:100) and incubated overnight at 4 °C to prepare the immune complex. The samples were washed with lysis buffer 3 times. Protein G Sepharose beads were then added, followed by further incubation for 1 h. The immunoprecipitates were washed 3 times with 1 mL of lysis buffer, eluted with reducing sample buffer, separated by SDS–PAGE and further analyzed by Western blotting.

### Transfection with miRNA

Transfection was performed using Lipofectamine 2000 Transfection Reagent (Invitrogen, 11668-019) according to the manufacturer’s instructions. HASMCs (5 × 10^4^ cells/well in a 6-well plate) were transfected with 20 µM siRNA by Lipofectamine 2000. After 24 h, the transfection mixture was removed and replaced with fresh culture media (Supplementary Table 2). The cells were cultured for 48 h prior to harvest.

### Statistical analysis

SPSS software (version 19.0, Inc., USA) was used for statistical analysis. For all tests, *P* < 0.05 was considered to indicate statistical significance. Independent t tests were used for comparisons between two groups. ANOVA was used for comparisons among multiple groups of data.

## Results

### Cell necrosis was significantly increased in patients with AAAD

**Fig. 1:**
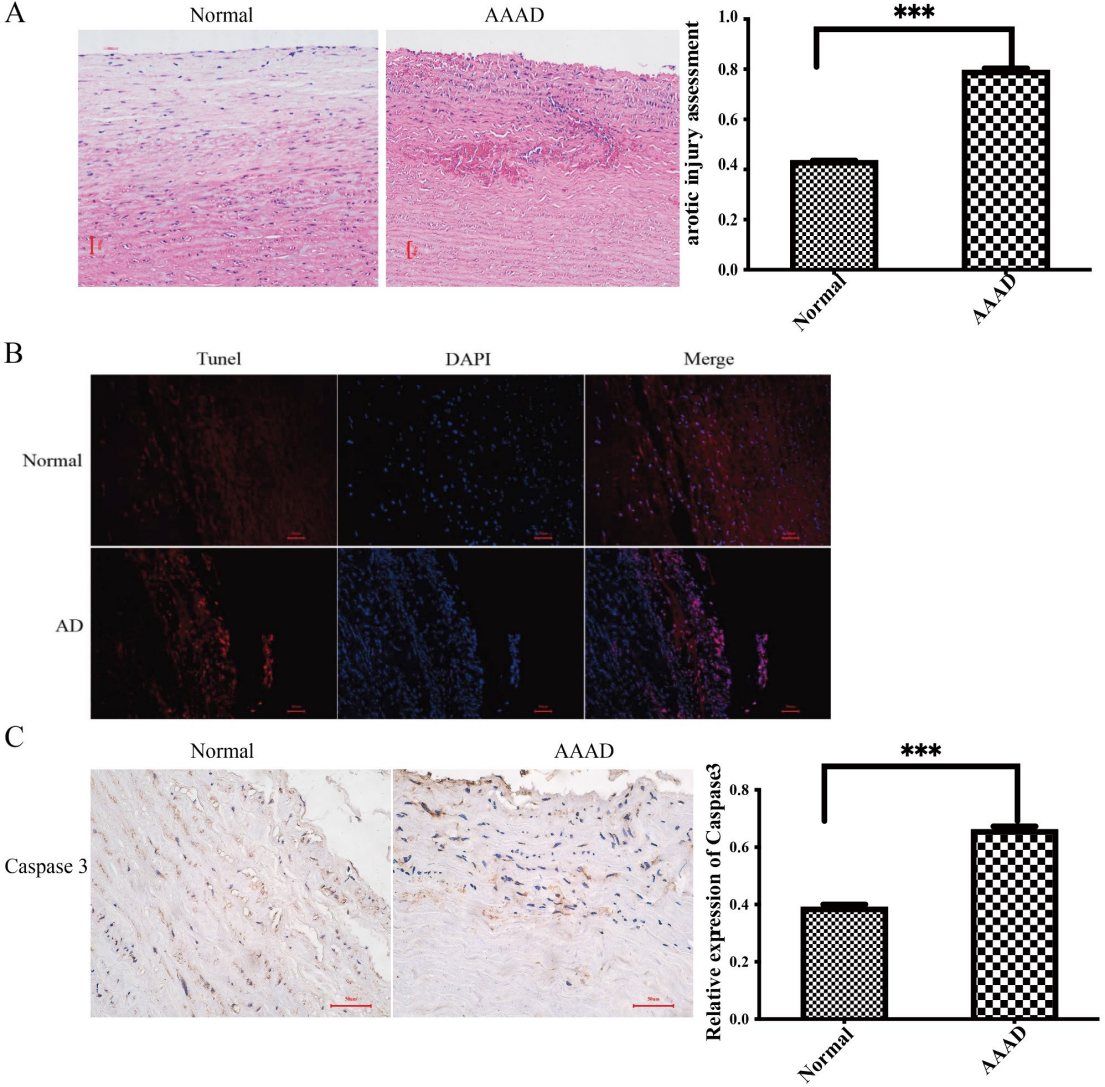
Pathological changes in patients with AAAD. **(A)** HE staining (original magnification ×100) showed that aortic apoptosis was significantly greater in the patients with AAAD. **(B)** The TUNEL assay showed that apoptosis in the patients with AAAD was significantly greater than that in normal aortas. **(C)** The level of Caspase 3 was increased significantly in the patients with AAAD (*P* < 0.05). (*n* = 8 patients in the AAAD group). ***P < 0.001 vs. the control

Hematoxylin and eosin (HE) staining indicated the degree of aortic pathological change in patients with AAAD (Fig. 1A). TUNEL staining was used to detect apoptosis in aortic tissue, and the results demonstrated that the level of aortic apoptosis in the patients with AAAD was significantly greater than that in normal aortas (Fig. 1B, C).

### The expression levels of integrin α5β1 decreased in samples from the patients with AAAD

**Fig. 2:**
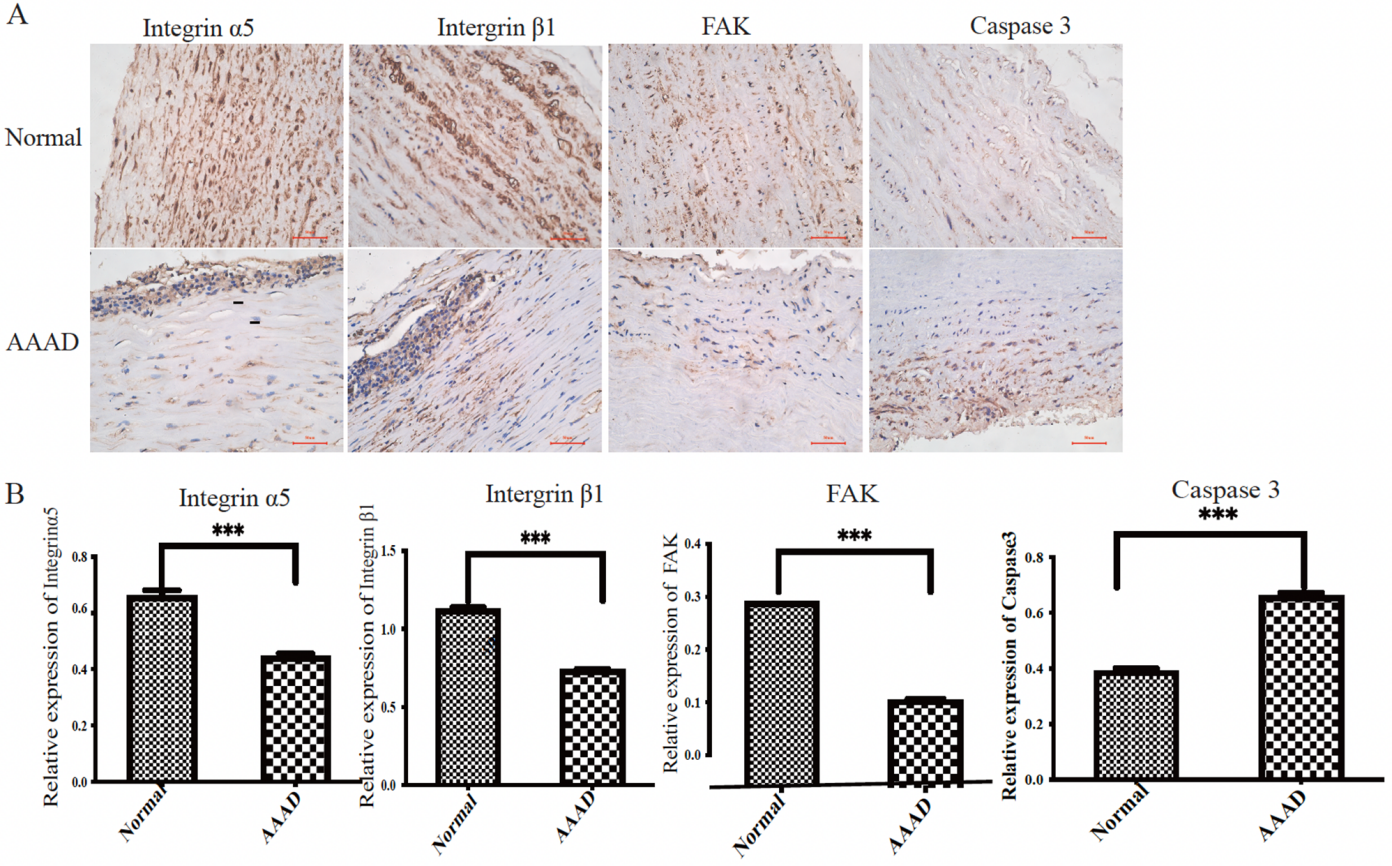
The expression levels of integrin α5, integrin β1 and FAK **(A)**. **(B)** Statistical analysis of the integrin α5, integrin β1 and FAK levels (*n* = 8 patients in the AAAD group). ***P < 0.001 vs. the control(*n* = 8 patients in the AAAD group). ***P < 0.001 vs. the control

We collected samples from the patients with AAAD and the corresponding controls. The protein expression levels of integrin α5β1 and FAK were determined via immunohistochemistry. In the patients with AAAD, the levels of integrin α5, integrin β1 and FAK were significantly lower and the level of Caspase 3 was significantly greater than those in the controls (*P* < 0.05) (Fig. 2A, B).

### The integrin α5β1-FAK axis affects the function of SMCs

**Fig. 3:**
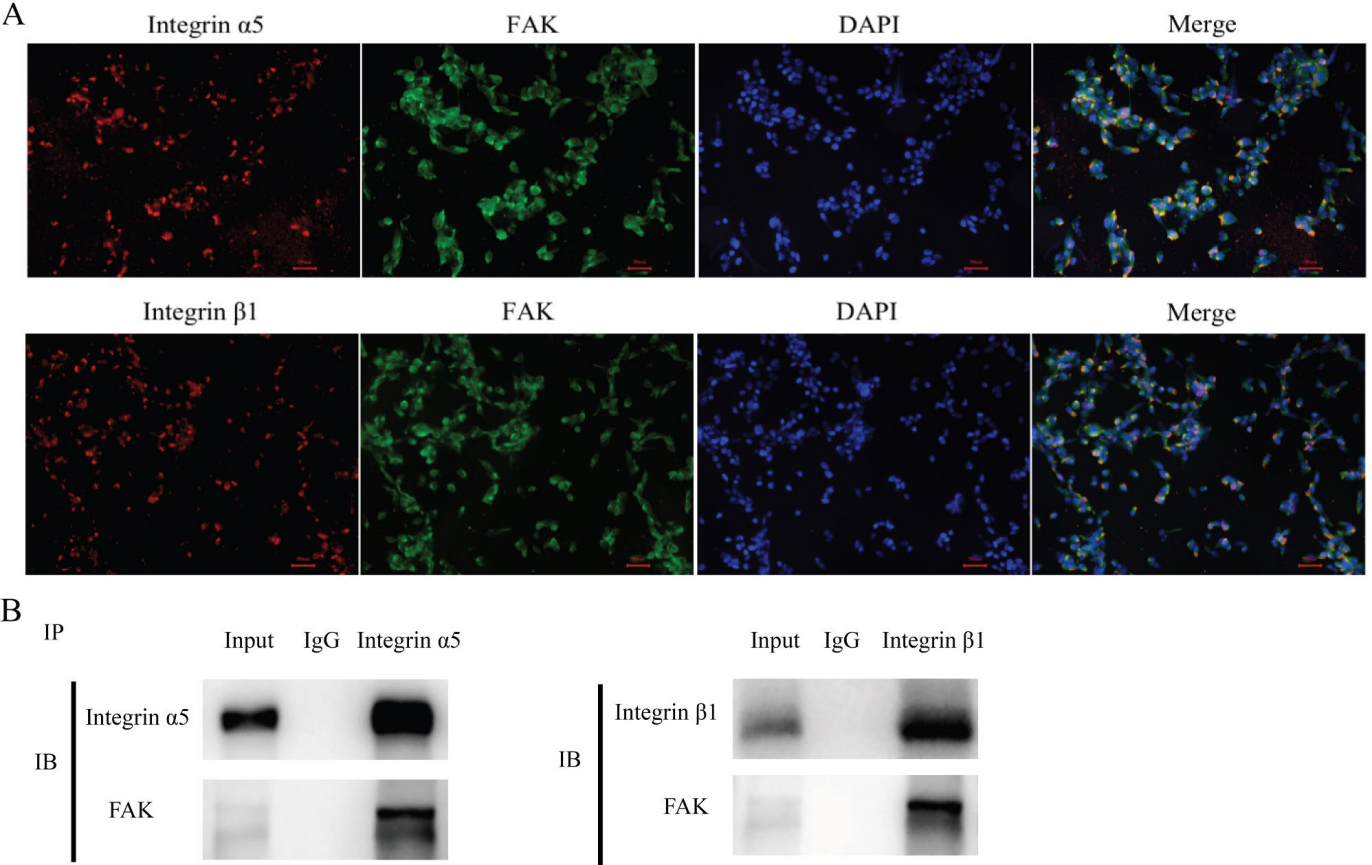
IF confirmed that integrin α5, integrin β1 and FAK are expressed in the same locations in HASMCs (200X) **(A)**. Integrin α5β1 affects cell function by binding with FAK in HASMCs **(B)**. (*n* = 3 per group)

In HASMCs, integrin α5, integrin β1 and FAK were simultaneously assessed via IF. We found that integrin α5β1 and FAK were expressed in the same region (Fig. 3A).

We next investigated whether integrin α5β1 affects the SMCs function by binding FAK protein. The immunoprecipitation results confirmed integrin α5 and FAK protein binding and integrin β1 and FAK protein binding (Fig. 3B).

### Downregulation of integrin α5β1 expression increased HASMC apoptosis

After the HASMCs were transfected with integrin α5β1 siRNA for 48 h, the cells in each group were collected, and integrin α5 and integrin β1 were assessed via qPCR. The best integrin α5 siRNA was siRNA-615, which resulted in the lowest integrin α5 mRNA expression level compared with that of the control group (Supplementary Fig. 1A). Furthermore, the best integrin β1 siRNA was siRNA-2504, which resulted in the lowest integrin β1 mRNA expression level compared with that of the control group (Supplementary Fig. 1B).

Supplementary Fig. 1 Detection of the mRNA expression levels of integrin α5 and integrin β1 in HASMCs by qPCR. A: Integrin α5 mRNA in HASMCs; B: Integrin β1 mRNA in HASMCs (*n* = 3 per group). **P < 0.01 vs. the control; ***P < 0.001 vs. the control.

**Fig. 4:**
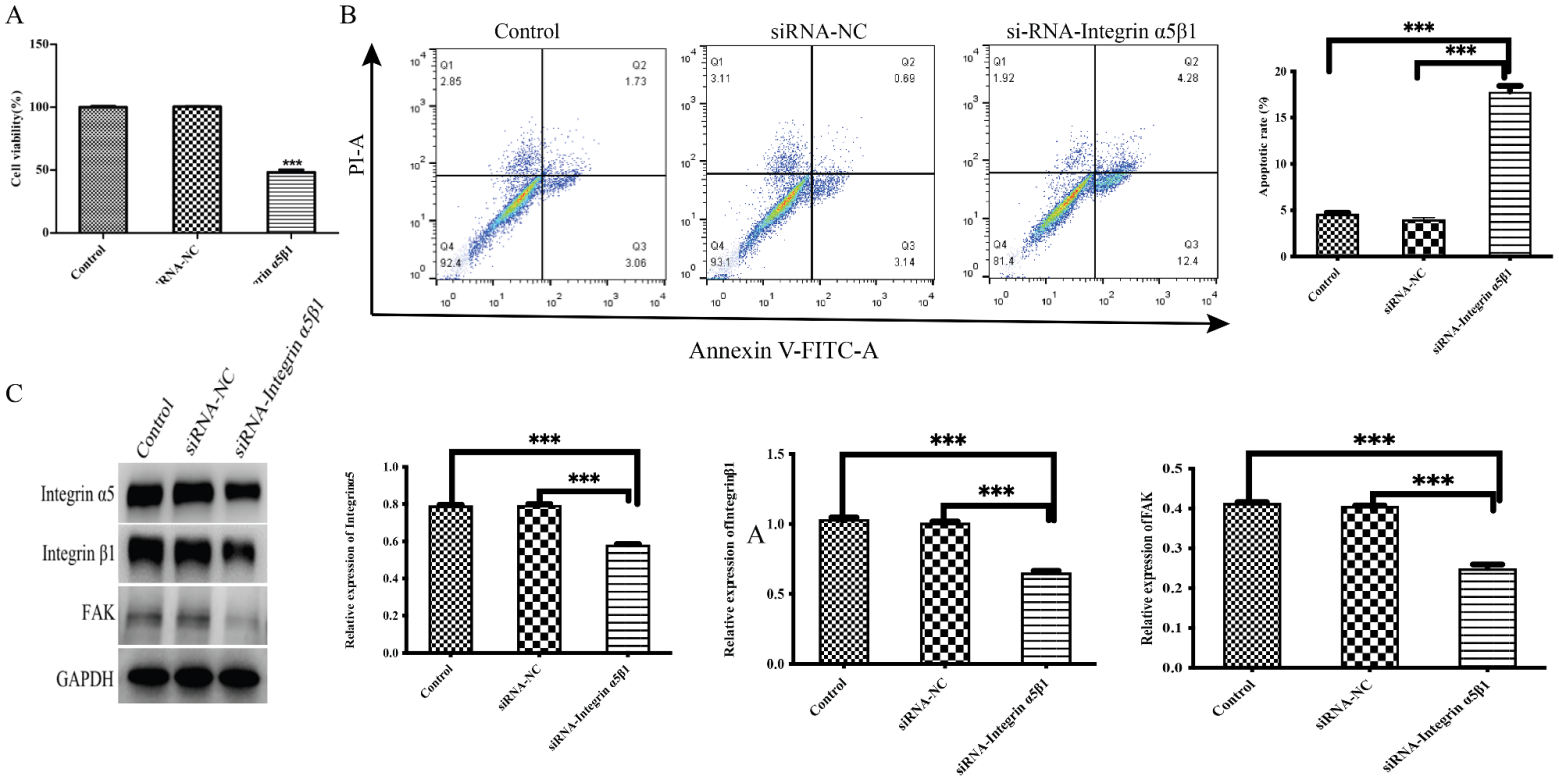
After HASMCs were transfected with integrin α5β1 siRNA for 48 h, cell viability decreased significantly **(A)**. **(B)** Compared with that in the control group, the percentage of apoptotic HASMCs increased following integrin α5β1 knockdown. **(C)** Western blot analysis confirmed that after integrin α5β1 was knocked down in HASMCs, the expression levels of integrin α5, integrin β1 and FAK decreased. ***P < 0.001 vs. the control *n* = 3 per group

After HAMSCs were transfected with integrin α5β1 siRNA for 48 h, CCK-8 assays were used to determine the survival rate of each group of cells. Compared with that of the control group, the viability of the HASMCs was significantly lower (*P* < 0.001) (Fig. 4A). The percentage of apoptotic cells in each group was determined via flow cytometry. Compared with that in the control group, the percentage of apoptotic HASMCs was significantly greater (*P* < 0.001) (Fig. 4B). The protein expression levels were determined by Western blotting. Compared with those in the control cells, the protein expression levels of integrin α5, integrin β1 and FAK in HASMCs were significantly lower following integrin α5β1 knockdown (*P* < 0.001) (Fig. 4C).

## Discussion

In this study, we demonstrated that the expression level of integrin α5β1 was significantly decreased in patients with AAAD and that this protein plays an important role in HASMCs. We found that the expression level of integrin α5β1 was decreased in patients with AAAD. Based on these results, we speculate that integrin α5β1 may be involved in the pathogenesis of AAAD. Integrin α5 and integrin β1 were downregulated by siRNA in HASMCs. After the knockdown of integrin α5 and β1, HASMCs exhibited decreased proliferation and increased apoptosis. These findings may be due to integrin α5β1 and FAK promoting the development of AAAD.

Recently, integrin α5β1 has been reported to be involved in vascular diseases. One study suggested that the activity of integrin α5β1 is decreased in the aortic tissue of abdominal aortic aneurysms, which is consistent with our findings [16]. Decreased integrin α5β1 activity may alter the structure of the abdominal aortic mesothelial layer, leading to aortic malformation and abdominal aortic aneurysms. Blocking the expression of integrin α5β1 in porcine carotid SMCs significantly reduced cell proliferation [17]. Similarly, in rat SMCs, the integrin α5β1 could enhance the adhesion, migration and proliferation of SMCs [18]. In the ECs of C57BL/6 mice, which do not express integrin α5β1, this molecule plays an important role in angiogenesis by promoting the proliferation of brain ECs in response to cerebral hypoxia [19]. These findings confirm that integrin α5β1 plays a potential pathological role in vascular diseases, including AAAD.

The integrin α5β1 plays an important role in angiogenesis and metastasis in many tumors. For example, integrin α5 and integrin β1 may mediate the initial adhesion process of the ovarian cancer integrin α5β1 [20]; targeting integrin α5β1 in ovarian cancer cell lines could inhibit the peritoneal dissemination of tumors [21]. In cholangiocarcinoma (CCA), integrin α5β1 could induce ductal epithelial–mesenchymal transformation; knockout of integrin α5β1 in CCA cells inhibited their migration [22]. In an in vivo experimental glioma model, upregulation of integrin α5β1 expression promoted tumor angiogenesis in glioblastoma cells [23]. Integrin α5β1 may also be associated with liver metastasis in melanoma. Given the above evidence, this protein is a promising target for the treatment of advanced cancer.

FAK, a nonreceptor tyrosine kinase, can induce the transduction of extracellular cues to intracellular signaling pathways via integrin α5β1 [24]. The integrin α5β1-FAK signaling pathway contributes to the progression of several diseases [25–27]. In this study, the integrin α5β1-FAK axis was shown to affect the function of SMCs. Integrin α5β1 affects the phosphorylation level of FAK. One study revealed that FAK is inactive in the SMCs of healthy arteries. However, vessel injury promotes FAK activation at integrin α5β1 adhesion sites and affects VSMC proliferation [24, 28]. Targeting the integrin α5β1-FAK axis could have therapeutic potential.

### Limitations

This study has several important limitations. First, because it is very difficult to obtain normal aortic tissue, control aortic tissue was obtained from patients who died after a traffic accident. Second, the aortic samples we studied represent end-stage disease, and we were not able to study changes in the early stages of AAAD. Third, despite the exclusion criteria, cardiovascular disease cannot be completely ruled out in the control group. Fourth, animal models of AAAD are needed to validate the conclusions of this study.

## Conclusion

In conclusion, this report demonstrated that integrin α5β1 participates in the pathogenesis of AAAD, and its downregulation may lead to an increased incidence of this disease.

### Electronic supplementary material

Below is the link to the electronic supplementary material.

**Supplementary Material 1: Supplementary Fig. 1.** Detection of the mRNA expression levels of integrin α5 and integrin β1 in HASMCs by qPCR. A: Integrin α5 mRNA in HASMCs; B: Integrin β1 mRNA in HASMCs (*n* = 3 per group). **P < 0.01 vs. the control; ***P < 0.001 vs. the control

**Supplementary Material 2: Supplementary Table 2.** The sequences of the siRNAs used in this study

**Supplementary Material 3: Figure 3.** Integrin α5 original image

**Supplementary Material 4: Figure 3.** Integrin β1 original image

**Supplementary Material 5: Figure 3.** FAK original image

**Supplementary Material 6: Figure 4.** Integrin α5 original image

**Supplementary Material 7**

**Supplementary Material 8: Figure 4.** FAK original image

**Supplementary Material 9: Figure 4.** GAPDH original image

**Supplementary Material 10: Supplementary Table 1a.** Demographic characteristics of the 8 patients with AAAD

**Supplementary Material 11: Supplementary Table 1b.** Genetic characteristics of the 8 patients with AAAD

**Supplementary Material 12:** All full-length gels and blot images

## Data Availability

The dataset is available from the corresponding author upon reasonable request.

## Abbreviations

AAAD: Acute type A aortic dissection
HASMC: Human aortic smooth muscle cell
SMC: Smooth muscle cell
FAK: Focal adhesion kinase
VEGFR2: Vascular endothelial growth factor 2
TUNEL: Terminal-deoxynucleotidyl transferase-mediated nick end labeling
EC: Endothelial cell
IHC: Immunohistochemical
IF: Immunofluorescence
CCK-8: Cell Counting Kit-8
OD: Optical density
HE: Hematoxylin-eosin staining
CCA: Cholangiocarcinoma
