# Supplementary material for: A decrease in integrin α5β1/FAK is associated with increased apoptosis of aortic smooth muscle cells in acute type a aortic dissection
Source: BMC Cardiovasc Disord. 2024 Mar 26;24:180. doi: 10.1186/s12872-024-03778-2 (PMC10964683; doi:10.1186/s12872-024-03778-2)
Supplement: Supplementary file 2 — Supplementary Material 2: Supplementary Table 2. The sequences of the siRNAs used in this study [file 12872_2024_3778_MOESM2_ESM.docx]

|  | Sequence（5'-3'） | antisense（5'-3'） |
| --- | --- | --- |
| siRNA-NC | UUCUCCGAACGUGUCACGUTT | ACGUGACACGUUCGGAGAATT |
| siRNA-615 | CACCCGAAUUCUGGAGUAUTT | AUACUCCAGAAUUCGGGUGTT |
| siRNA-733 | GGACCAGGAAGCUAUUUCUTT | AGAAAUAGCUUCCUGGUCCTT |
| siRNA-1329 | GCAGGGAGUAGUGUUUGUATT | UACAAACACUACUCCCUGCTT |
| siRNA-NC | UUCUCCGAACGUGUCACGUTT | ACGUGACACGUUCGGAGAATT |
| siRNA-474 | GGCUCCAAAGAUAUAAAGATT | UCUUUAUAUCUUUGGAGCCTT |
| siRNA-2172 | GCCUUCAAUAAAGGAGAAATT | UUUCUCCUUUAUUGAAGGCTT |
| siRNA-2504 | GGAGUUUGCUAAAUUUGAATT | UUCAAAUUUAGCAAACUCCTT |

**Supplementary Table 2 The sequence of siRNA in this study**
