# Supplementary figures and images for: A decrease in integrin α5β1/FAK is associated with increased apoptosis of aortic smooth muscle cells in acute type a aortic dissection
Source: BMC Cardiovasc Disord. 2024 Mar 26;24:180. doi: 10.1186/s12872-024-03778-2 (PMC10964683; doi:10.1186/s12872-024-03778-2)

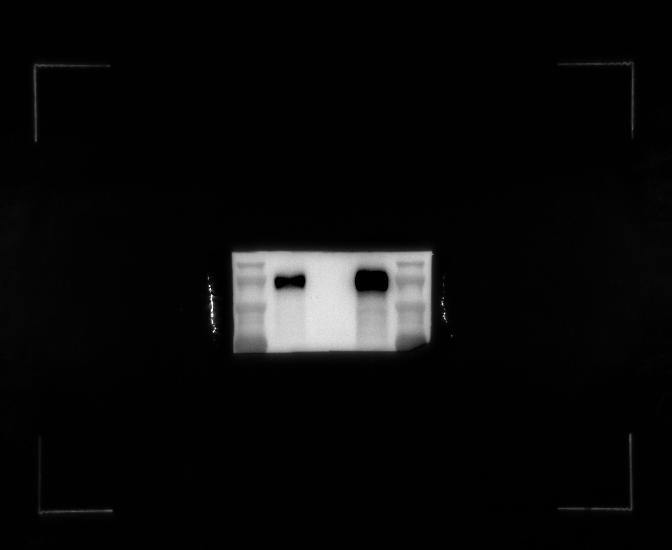

Supplement: Supplementary file 3 — Supplementary Material 3: Figure 3. Integrin α5 original image [file 12872_2024_3778_MOESM3_ESM.tif]

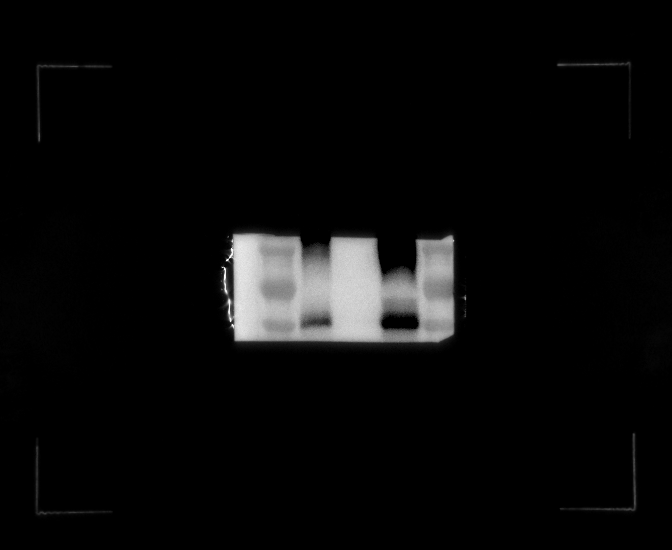

Supplement: Supplementary file 4 — Supplementary Material 4: Figure 3. Integrin β1 original image [file 12872_2024_3778_MOESM4_ESM.tif]

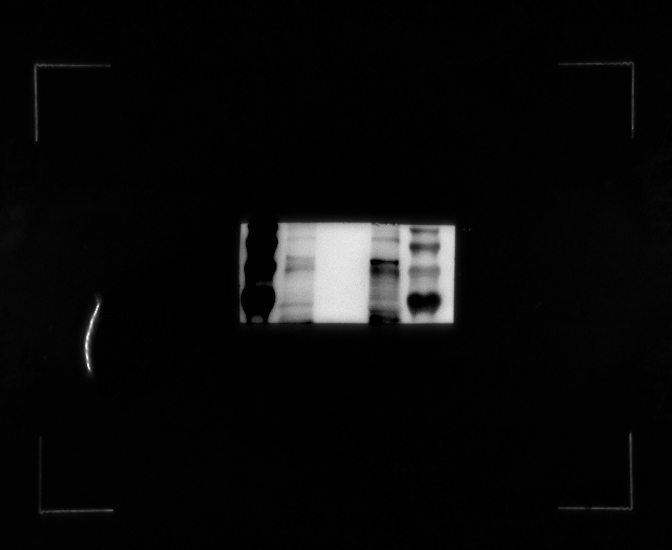

Supplement: Supplementary file 5 — Supplementary Material 5: Figure 3. FAK original image [file 12872_2024_3778_MOESM5_ESM.tif]

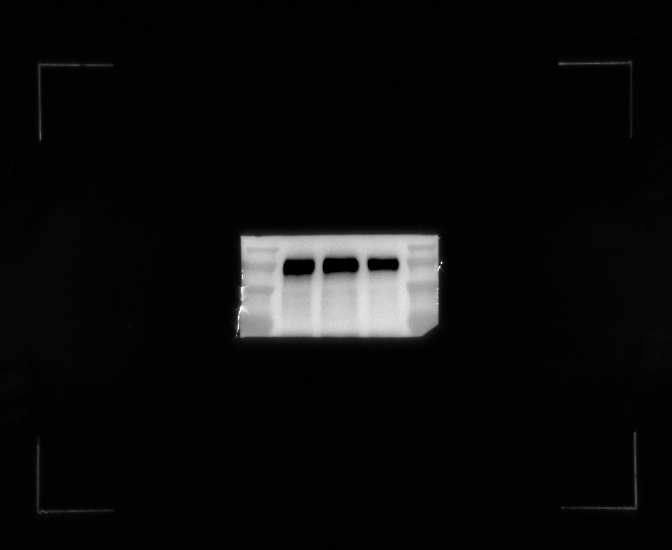

Supplement: Supplementary file 6 — Supplementary Material 6: Figure 4. Integrin α5 original image [file 12872_2024_3778_MOESM6_ESM.tif]

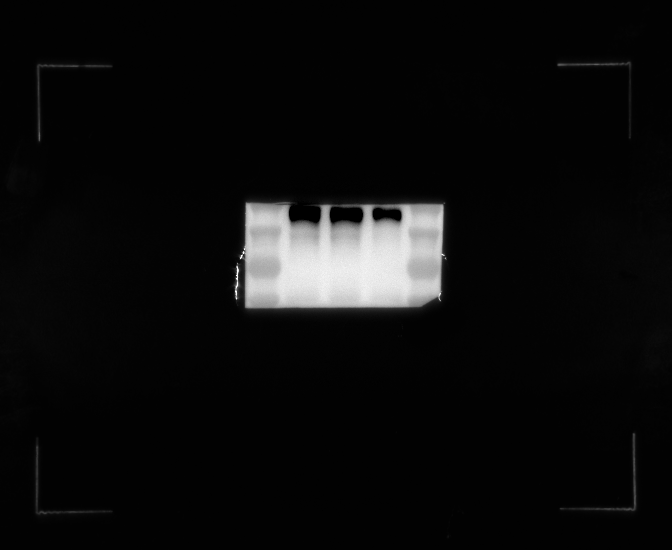

Supplement: Supplementary file 7 — Supplementary Material 7 [file 12872_2024_3778_MOESM7_ESM.tif]

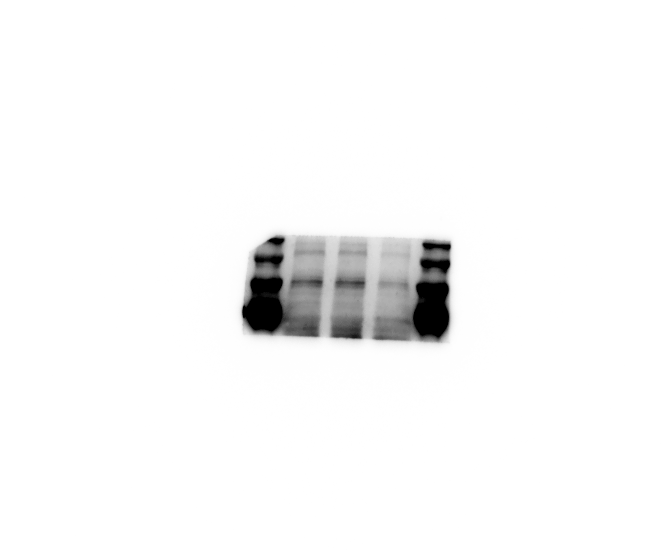

Supplement: Supplementary file 8 — Supplementary Material 8: Figure 4. FAK original image [file 12872_2024_3778_MOESM8_ESM.tif]

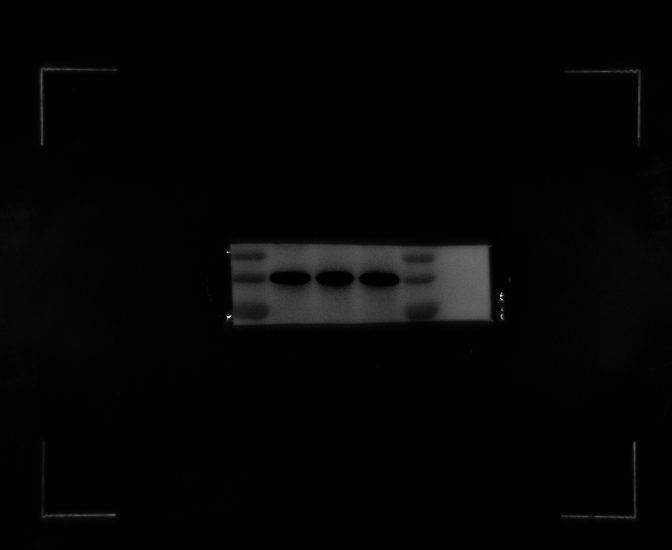

Supplement: Supplementary file 9 — Supplementary Material 9: Figure 4. GAPDH original image [file 12872_2024_3778_MOESM9_ESM.tif]
