# Supplementary material for: A decrease in integrin α5β1/FAK is associated with increased apoptosis of aortic smooth muscle cells in acute type a aortic dissection
Source: BMC Cardiovasc Disord. 2024 Mar 26;24:180. doi: 10.1186/s12872-024-03778-2 (PMC10964683; doi:10.1186/s12872-024-03778-2)
Supplement: Supplementary file 10 — Supplementary Material 10: Supplementary Table 1a. Demographic characteristics of the 8 patients with AAAD [file 12872_2024_3778_MOESM10_ESM.docx]

|  | AAAD1 | AAAD2 | AAAD3 | AAAD4 | AAAD5 | AAAD6 | AAAD7 | AAAD8 |
| --- | --- | --- | --- | --- | --- | --- | --- | --- |
| Sex | Male | Male | Male | Female | Male | Male | Female | Female |
| Age (y) | 41 | 65 | 55 | 51 | 56 | 59 | 54 | 60 |
| Dissection characteristics |  |  |  |  |  |  |  |  |
| Diagnosis and comments | AAAD | AAAD | AAAD | AAAD | AAAD | AAAD | AAAD | AAAD |
| Aortic diameter (cm) | 48 | 52 | 49 | 48 | 53 | 51 | 50 | 52 |
| Time from symptom onset to surgery (h) | 23 h | 5 h | 24 h | 8 h | 7 h | 16.5 h | 11 h | 14 h |
| Patient history |  |  |  |  |  |  |  |  |
| Known aortic aneurysm | NO | NO | NO | NO | NO | NO | NO | NO |
| Bicuspid aortic valve | NO | NO | NO | NO | NO | NO | NO | NO |
| Atherosclerosis | NO | NO | NO | NO | NO | NO | NO | NO |
| Prior cardiac surgery (any) | NO | NO | NO | NO | NO | NO | NO | NO |
| Smoking status | NO | NO | NO | NO | NO | NO | NO | NO |
| Diabetes mellitus | NO | YES | NO | NO | NO | NO | NO | NO |
| Hypertension | YES | YES | YES | YES | YES | YES | YES | YES |
| Chronic obstructive pulmonary disease | NO | NO | NO | NO | NO | NO | NO | NO |
| History of aortic valve replacement | NO | NO | NO | NO | NO | NO | NO | NO |
| History of mitral valve replacement | NO | NO | NO | NO | NO | NO | NO | NO |
| History of coronary artery bypass graft | NO | NO | NO | NO | NO | NO | NO | NO |
| History of aneurysm/acute aortic dissection surgery | NO | NO | NO | NO | NO | NO | NO | NO |
| Marfan syndrome | NO | NO | NO | NO | NO | NO | NO | NO |
| Sphrintzene-Goldberg syndrome | NO | NO | NO | NO | NO | NO | NO | NO |
| Loeyse-Dietz syndrome | NO | NO | NO | NO | NO | NO | NO | NO |
| Vascular Ehlerse-Danlos syndrome | NO | NO | NO | NO | NO | NO | NO | NO |

**Supplementary Table 1a Demographic characteristics of AAAD patients**
