# Supplementary material for: A decrease in integrin α5β1/FAK is associated with increased apoptosis of aortic smooth muscle cells in acute type a aortic dissection
Source: BMC Cardiovasc Disord. 2024 Mar 26;24:180. doi: 10.1186/s12872-024-03778-2 (PMC10964683; doi:10.1186/s12872-024-03778-2)
Supplement: Supplementary file 11 — Supplementary Material 11: Supplementary Table 1b. Genetic characteristics of the 8 patients with AAAD [file 12872_2024_3778_MOESM11_ESM.docx]

| Supplementary Table 1b. The genetic evaluation of the 8 AAAD patients | | | | | | | | |
| --- | --- | --- | --- | --- | --- | --- | --- | --- |
| Variable | AAAD1 | AAAD2 | AAAD3 | AAAD4 | AAAD5 | AAAD6 | AAAD7 | AAAD8 |
| **Marfan syndrome** |  |  |  |  |  |  |  |  |
| Family history of Marfan syndrome | No | No | No | No | No | No | No | No |
| System scores | 0 | 0 | 0 | 0 | 0 | 0 | 0 | 0 |
| Wrist sign + thumb sign | No | No | No | No | No | No | No | No |
| chicken breast deformity | No | No | No | No | No | No | No | No |
| hindfoot deformity | No | No | No | No | No | No | No | No |
| pneumothorax | No | No | No | No | No | No | No | No |
| dural dilatation | No | No | No | No | No | No | No | No |
| acetabular impingement disease | No | No | No | No | No | No | No | No |
| Decreased upper segment/lower segment (US/LS) ratio and increased arm span/height ratio without severe scoliosis | No | No | No | No | No | No | No | No |
| Scoliosis or thoracolumbar kyphosis | No | No | No | No | No | No | No | No |
| Decreased elbow abduction | No | No | No | No | No | No | No | No |
| Facial features [at least 3 of the following 5 features: long head deformity (reduced cephalic index or reduced head width/length ratio), sunken eyes, downward sloping eyelid fissures, zygomatic dysplasia, and retruded jaws] | No | No | No | No | No | No | No | No |
| leather strap | No | No | No | No | No | No | No | No |
| Myopia greater than 3 diopters | No | No | No | No | No | No | No | No |
| All types of MVPs | No | No | No | No | No | No | No | No |
| Aortic root diameter Z score | 6.22 | 5.04 | 5.24 | 6.85 | 5.3 | 6.03 | 5.4 | 6.86 |
| Male/Female | M | M | M | F | M | F | M | F |
| Height(cm) | 172 | 169 | 175 | 162 | 171 | 163 | 173 | 159 |
| Weight(kg) | 72 | 73 | 75.5 | 62.4 | 73.7 | 67.5 | 78.5 | 63 |
| Age(Years) | 41 | 65 | 55 | 51 | 56 | 71 | 50 | 51 |
| Ao Root at sinuses of Valsalva(in cm) | 5 | 4.9 | 4.8 | 5.1 | 4.8 | 5.2 | 5.1 | 5.05 |
| Gene Detection |  |  |  |  |  |  |  |  |
| FBN1 | **N** | **N** | **N** | **N** | **N** | **N** | **N** | **N** |
| TGFBR1/2 | **N** | **N** | **N** | **N** | **N** | **N** | **N** | **N** |
| Sphrintzene-Goldberg syndrome | No | No | No | No | No | No | No | No |
| Loeyse-Dietz syndrome | No | No | No | No | No | No | No | No |
| Vascular Ehlerse-Danlos syndrome | No | No | No | No | No | No | No | No |
| AAAD,acute type A aortic dissection |  |  |  |  |  |  |  |  |
