# Supplementary material for: A decrease in integrin α5β1/FAK is associated with increased apoptosis of aortic smooth muscle cells in acute type a aortic dissection
Source: BMC Cardiovasc Disord. 2024 Mar 26;24:180. doi: 10.1186/s12872-024-03778-2 (PMC10964683; doi:10.1186/s12872-024-03778-2)

All full-length gels and blot images

1 Figure3-FAK original image


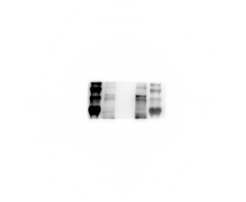


2 Figure3-Integrin α5 original image


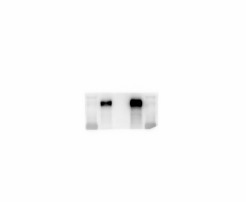


3 Figure3-Integrin β1 original image


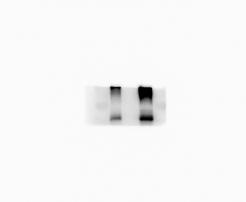


4 Figure4-FAK original image


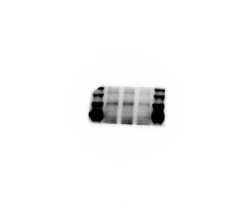


5 Figure4-GAPDH original image


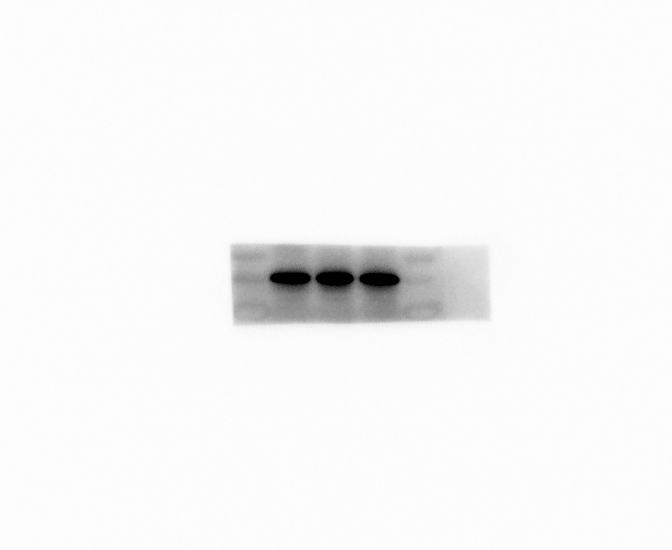


6 Figure4-Integrin α5 original image


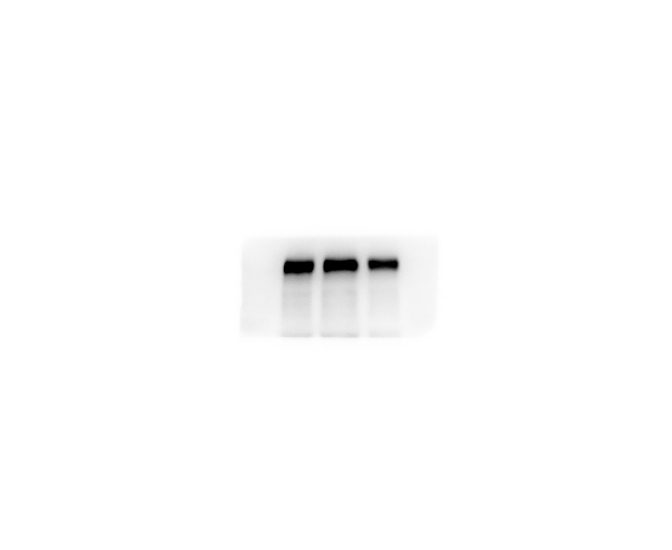

Supplement: Supplementary file 12 — Supplementary Material 12: All full-length gels and blot images [file 12872_2024_3778_MOESM12_ESM.docx]
